# A Unique Case of Primary Ewing's Sarcoma of the Cervical Spine in a 53-Year-Old Male: A Case Report and Review of the Literature

**DOI:** 10.1155/2015/402313

**Published:** 2015-02-23

**Authors:** Marshall T. Holland, Oliver E. Flouty, Liesl N. Close, Chandan G. Reddy, Matthew A. Howard

**Affiliations:** Department of Neurosurgery, University of Iowa, 200 Hawkins Drive, Iowa City, IA 52246, USA

## Abstract

Extraskeletal Ewing's sarcoma (EES) is a rare presentation, representing only 15% of all primary Ewing's sarcoma cases. Even more uncommon is EES presenting as a primary focus in the spinal canal. These rapidly growing tumors often present with focal neurological symptoms of myelopathy or radiculopathy. There are no classic characteristic imaging findings and thus the physician must keep a high index of clinical suspicion. Diagnosis can only be definitively made by histopathological studies. In this report, we discuss a primary cervical spine EES in a 53-year-old man who presented with a two-month history of left upper extremity pain and acute onset of weakness. Imaging revealed a cervical spinal canal mass. After undergoing cervical decompression, histopathological examination confirmed a diagnosis of Ewing's sarcoma. A literature search revealed fewer than 25 reported cases of primary cervical spine EES published in the past 15 years and only one report demonstrating this pathology in a patient older than 30 years of age (age = 38). Given the low incidence of this pathology presenting in this age group and the lack of treatment guidelines, each patient's plan should be considered on a case-by-case basis until further studies are performed to determine optimal evidence based treatment.

## 1. Introduction

Ewing's sarcoma is a malignant primitive neuroectodermal tumor (PNET) that primarily presents during the first two decades of life. Approximately 85% of those cases present primarily in the skeleton. With an annual incidence of 1–3 per million, it is generally a rare diagnosis [1]. EES occurs most often during the second or third decade of life. While the spinal canal remains a rare site for Ewing's sarcoma to arise, more common sites for an EES to present include the head and neck, buttocks, lower extremities, chest wall, and retroperitoneal space [2]. There have been examples of both intradural and extradural presentations of primary spinal EES [3, 4]. Primary spinal EES has a predilection for the sacral spine, with 50% of spinal EES appearing in this location [5]. Although reported, it is unusual to see cervical spine involvement in a primary EES [5, 6]. Acute to subacute localizable pain, myelopathic symptoms, and radicular symptoms are common presenting complaints. Upon performing a PubMed Search for English language articles on EES, we found less than 25 reported cases of EES with at least partial involvement of the cervical spine in the past 15 years. All of these have occurred in patients less than 40 years of age [5, 7–21].

## 2. Case Report

### 2.1. History and Physical Examination

A 53-year-old male first presented to his primary care provider with a 2-month history of progressive complaints that started as a stiff neck and subjective right shoulder blade mass that was never objectively noted by any physician with some right upper extremity pain. This pain reportedly then spread to the left upper extremity with pain radiating down the posterior side to the left proximal wrist. Due to these complaints, he was evaluated by his primary care provider and started on ibuprofen. Upon awakening the following morning, the patient reported the sudden onset of left arm weakness that was not antigravity. Conversely however, the left upper extremity pain had remitted. With this sudden change in neurological function, the patient's primary care provider obtained an MRI of the cervical spine. The patient was told that the MRI showed a bony lesion and he was referred to our institution's orthopedics clinic. One month later he presented to the orthopedic clinic where the patient noted that his left upper extremity pain had returned. The patient and his wife also noted that, over the previous two-month period, he was losing muscle mass most profoundly in the left biceps and deltoid. Furthermore, he had also unintentionally lost 10 pounds over the previous three months. Due to his suspicious presentation and cervical spine lesion noted on the MRI, the patient was referred to our neurosurgical clinic.

**Figure 1:**
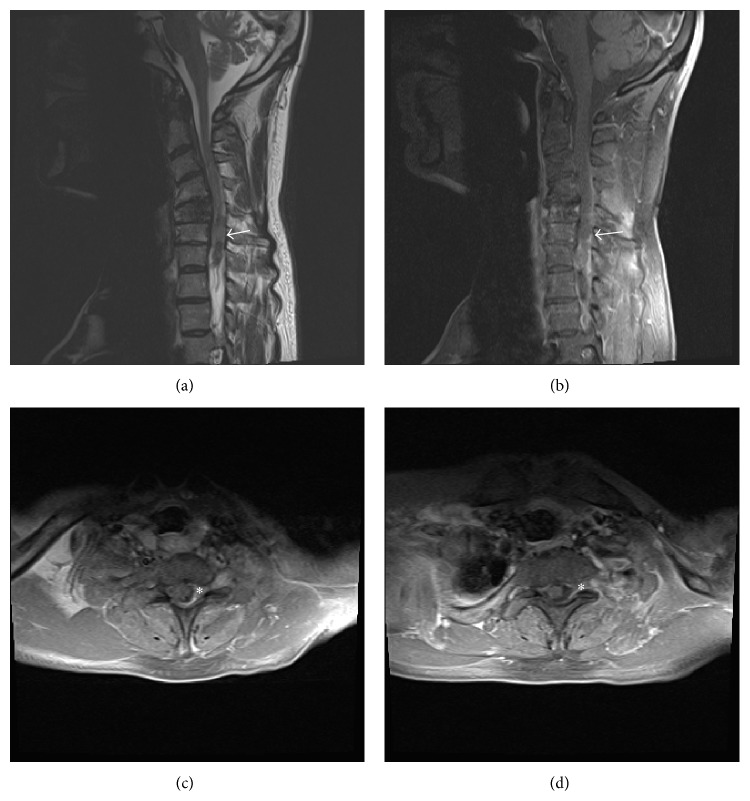
Cervical MRI: (a) midsagittal T2 MRI (TR = 4000, TE = 98) and (b) midsagittal T1 postcontrast (TR = 659, TE = 8.7) imaging of the cervical spine showing a C2–C7 ill-defined extradural mass that is hypointense on T2 and enhances with contrast most predominant at C6-C7. ((c) and (d)) Two axial slices of a preoperative T1 postgadolinium contrast (TR = 591, TE = 7.3) MRI of the cervical spine passing through the C6-C7 level demonstrates an extradural, enhancing mass invading the spinal canal eccentric to the left.

On examination, the patient appeared emaciated and easily fatigable. Muscle strength testing revealed 2/5 in the left upper extremity with exception of the biceps at 3/5 and handgrip of 4-/5. His right upper extremity was 3/5 in the deltoid and 4-/5 in all other muscle groups. The bilateral lower extremities were 5/5 for strength. There were no myelopathic signs. A repeat MRI performed at our institution showed an ill-defined predominantly extradural enhancement along the left aspect of the cervical spinal canal, most apparent at C6-C7, but also extending between C2 and C7 (Figure 1). A CT chest/abdomen/pelvis showed no evidence of other primary diseases. After being admitted to the hospital, the patient acutely developed increased difficulty with his gait and experienced an episode of urinary incontinence.

### 2.2. Operation

The patient underwent a C6-C7 laminotomy with decompression of the left C7 nerve root and biopsy of the extradural lesion, accomplishing the goals of operation including decompression and definitive diagnosis. Intraoperative baseline SSEPs were attempted; however no electrophysiological recordings could be obtained apart from minimal recordings in the right upper extremity.

### 2.3. Pathological Findings

**Figure 2:**
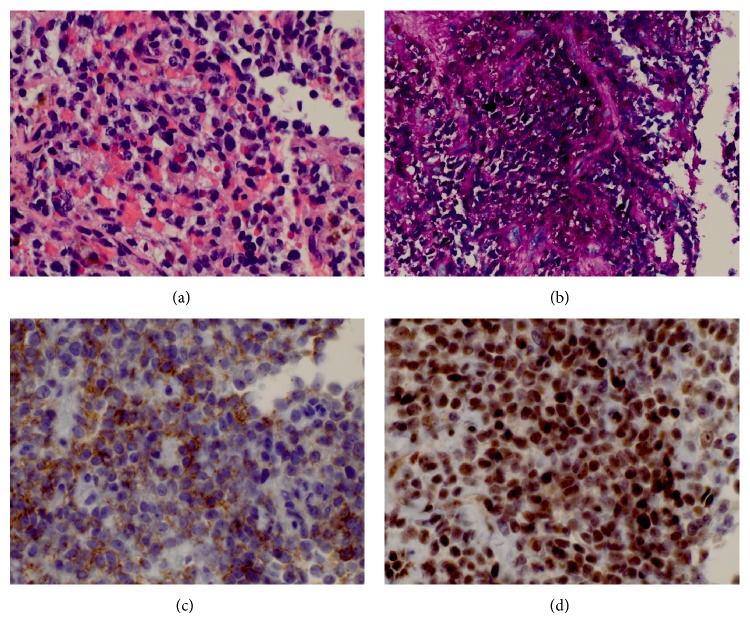
Histopathological investigation of the biopsied mass included (a) H&E staining that revealed round hyperchromatic nuclei with little cytoplasm and numerous mitoses. (b) Positive PAS staining of the tumor cells shows these cells to be glycogen rich. There is also positive staining for (c) CD-99 and (d) FLI-1.

Histopathological studies established the diagnosis of Ewing's sarcoma. Our pathologists noted round, hyperchromatic nuclei with little cytoplasm and multiple mitosis suggestive of a primitive neuroectodermal tumor in surrounding necrotic tissue (Figure 2(a)). Further histochemical staining showed that the tumor cells stained positively for PAS, CD-99 and FLI-1 (Figures 2(c) and 2(d)). The cells stained negative for pankeratin, S100, synatophysin, chromogranin CD45, CD5, and CD20. Following these immunohistochemical protocols, the diagnosis of Ewing's sarcoma was confirmed.

### 2.4. Postoperative Course

Upon awakening postoperatively, the patient was noted to be severally quadriparetic and had suffered a myocardial infarction. He was transferred to the surgical intensive care unit where he was kept on flat bed-rest and high mean arterial pressure control. He recovered remarkably well. First, he regained movement in his lower extremities and subsequently regained antigravity ability in his right upper extremity. He continued to improve with the help of physical and occupational therapy. At his 6-month follow-up appointment his muscle strength was slowly improving with 3/5 in the left upper extremity with exception of handgrip, which was 4-/5. His right upper extremity was 4-/5 throughout. The bilateral lower extremities were 4/5 in strength. He was started on a course of chemotherapy and radiation therapy prior to discharge. To date, he has completed radiation therapy with a total treatment of 55.8 Gy. He is currently undergoing infusion treatment sessions of vincristine, actinomycin, and cyclophosphamide alternating with ifosfamide and etoposide.

## 3. Discussion

Extraskeletal Ewing's sarcoma of the spinal canal remains a rare diagnosis. It has been a subject of interest since its first published description in 1969 [22]. EES affects males as often as females during the second or third decade of life, slightly later than the classic age of presentation for primary skeletal Ewing's sarcoma [8, 23]. The case presented here is a rare example of a primary extraskeletal Ewing's sarcoma of the cervical spinal canal. Furthermore, this case occurred in an adult patient in his sixth decade of life, far outside the typical presenting age range, 15 years older than any previously reported case. Ewing's sarcoma classically presents with local swelling and pain from the rapidly enlarging mass. When this occurs in the spinal canal, this can, as in our patient, initially present with pain and progress to other neurological symptoms such as weakness, numbness, tingling, or gait disturbances [5, 7, 8, 18, 24]. It is important to emphasize that these signs and symptoms can progress fairly rapidly, as in our case, warranting urgent surgical intervention [25].

In reviewing previously published case reports of primary EES of the cervical spine there were some patterns of presentation. The average age at presentation was 19.7 years and there were no cases identified that presented in a patient past 38 years of age. There was a slight propensity for the pathology to present in males (42%) compared to females (29%) in cases where gender was specified. However, one must take into account that 29% of the cases identified did not report the patient's gender. A further breakdown of these previous cases is summarized in Table 1.

Due to the rarity of this pathology, there are few large retrospective studies available for review. However, one such study performed by Ilaslan at the Mayo Clinic was able to identify 125 patients with primary spinal EES out of 1,277 patients with primary Ewing's sarcoma treated at the clinic from 1926 to 2001. The average age of the patients was 19.3 years (range 4–54 years). When the EES was organized by level in the spinal column, there were only a total of 4 patients identified (3%), further showing the infrequency of this pathology [5].

In terms of imaging, reports vary and there appears to be no classical findings of an EES mass. On CT, there tends to be a mass that enhances heterogeneously [18]. On MRI imaging the mass may appear hypo-, iso-, or hyperintense on T1 and hypo or hyperintense on T2. The mass often appears to enhance heterogeneously [8, 26]. Thus PNET should be included in the differential diagnosis of nonspecific spinal canal lesions. Our lesion was hypodense on T1 and slightly hyperintense on T2 and enhanced with contrast administration (Figure 1).

Definitive diagnosis is made by histopathological studies. Findings include uniform small round blue cells with scant possibly slightly eosinophilic cytoplasm notable on light microscopy. Two cellular markers frequently employed by pathologists are CD-99, which is expressed in both Ewing's sarcoma and lymphoma, and CD-45, which is only expressed in lymphoblastic lymphoma [1]. Typically, in order to confirm a diagnosis of Ewing sarcoma, the pathologist must rule out multiple other differentials through the process of multiple immunohistochemical staining beyond the scope of this paper [27].

Concerning treatment, there is strong evidence that early and aggressive treatment leads to the most favorable outcomes [8, 14, 17, 23]. In our case, there was strong evidence of a rapidly enlarging mass with rapidly progressive neurologic deficit. This warranted the first goal of operation to be decompression of the spinal cord with the proposed laminectomy. In other cases, if the mass was not producing rapidly progressive neurologic change, an argument may be made to first proceed with a needle biopsy and possible preoperative chemotherapy, shrinking the tumor and promoting an increased chance of successful gross total resection, followed by radiation therapy. Regardless, there is strong evidence that an aggressive multimodal treatment approach involving surgery, chemotherapy, and local radiation therapy increases the chance of a successful outcome [8, 17, 23].

Survivorship of primary EES of the spine post treatment was fairly consistent across different studies. A study by Venkateswaran from St. Jude's Children's Research Hospital of 33 patients showed an estimated 5-year survival rate of 48.1% (±8.9%) [28]. This was comparable to the Mayo Clinic study that showed a 5-year disease-free survival among its 125 patients at 45% for nonsacral lesions. Interestingly, this study also noted a 60% 5-year disease-free survival rate for sacral lesions [5]. One world review of the literature performed by an Italian group found various 5-year survival rates for primary spinal canal EES ranging from 0% to 37.5% [29, 30].

## 4. Conclusions

The occurrence of an extraskeletal Ewing's sarcoma remains rare, which typically affects adolescents with a male preponderance. It is especially rare to be found in the spine, especially the cervical spine. We have shown with this case report and in a review of prior literature that it is not unheard of for EES to present in the spine, regardless of age, and in our case, even in late adulthood. While it remains rare for an EES to present in the spine, it should remain a viable differential diagnosis of an ill-defined spinal mass noted on imaging, independent of the patient's age. Standard treatment often involves a multidisciplinary approach of surgery, chemotherapy, and radiation therapy with the particular preferential order determined on a case-by-case basis and has a survivorship of approximately 45% at 5 years after treatment.

## Figures and Tables

**Table 1:** Summary review: primary EES of cervical spine (available in English).

Authors	Number of cases	Year of publishing	Cervical level	Age/gender	Presenting symptoms
Martin Garcia et al. [14]	1	1991	C2	15/M	Right posterior neck pain

Sharafuddin et al. [17]	1	1992	C5	21/F	Neck pain and RUE radicular pain, progressive spastic quadriparesis

Villas and San Julian [19]	1	1996	C6	14/(n/a)	n/a

Kennedy et al. [8]	1	2000	C1–C5	24/M	Neck pain and neck mass

Mukhopadhyay et al. [15]	2	2001	C3–C5	29/F	Acute progressive quadriparesis and urinary retention
			C3–C5	13/M	Severe back pain

Shin et al. [18]	2	2001	C7–T1	22/F	Right arm paresthesia
			C5–C7	38/M	Right shoulder pain

Venkateswaran et al. [28]	2	2001	n/a	n/a	Local pain

Ilaslan et al. [5]	4	2004	n/a	n/a	Local pain

Kara [12]	1	2004	C5-C6	18/F	Back pain and fever

Kogawa et al. [13]	1	2004	C2–C4	7/F	Neck pain and LUE weakness

Bozkurt et al. [7]	1	2007	C2–C5	28/M	Neck pain and gait disturbance

Ozturk et al. [16]	1	2007	C6–T1	18/M	Right neck and shoulder pain with progression to paraplegia and urinary retention

Ali et al. [9]	1	2008	C6–T1	14/M	Neck mass

Bacci et al. [29]	2	2009	n/a	n/a	n/a

Hao et al. [11]	1	2010	C6	15/M	Neck/shoulder pain RUE numbness

Gulati et al. [21]	1	2011	C2	11/F	Nonradiating neck pain

Ellis et al. [31]	1	2011	C5–C7	27/M	Acute ascending weakness and sensory changes

Cabral et al. [10]	1	2012	C1-C2	22/F	Neck pain and right arm paresthesia

Current case	1	2013	C1–C7	53/M	LUE pain and weakness

	**26**				

n/a: not available.
